# Cdx2 regulates immune cell infiltration in the intestine

**DOI:** 10.1038/s41598-021-95412-w

**Published:** 2021-08-04

**Authors:** Simon Chewchuk, Sanzida Jahan, David Lohnes

**Affiliations:** Department of Cellular and Molecular Medicine, University of Ottawa, Ottawa, ON Canada

**Keywords:** Biochemistry, Cell biology, Genetics, Immunology, Microbiology, Stem cells

## Abstract

The intestinal epithelium is a unique tissue, serving both as a barrier against pathogens and to conduct the end digestion and adsorption of nutrients. As regards the former, the intestinal epithelium contains a diverse repertoire of immune cells, including a variety of resident lymphocytes, macrophages and dendritic cells. These cells serve a number of roles including mitigation of infection and to stimulate regeneration in response to damage. The transcription factor Cdx2, and to a lesser extent Cdx1, plays essential roles in intestinal homeostasis, and acts as a context-dependent tumour suppressor in colorectal cancer. Deletion of Cdx2 from the murine intestinal epithelium leads to macrophage infiltration resulting in a chronic inflammatory response. However the mechanisms by which Cdx2 loss evokes this response are poorly understood. To better understand this relationship, we used a conditional mouse model lacking all intestinal Cdx function to identify potential target genes which may contribute to this inflammatory phenotype. One such candidate encodes the histocompatability complex protein H2-T3, which functions to regulate intestinal iCD8α lymphocyte activity. We found that Cdx2 occupies the *H3-T3* promoter in vivo and directly regulates its expression via a Cdx response element. Loss of Cdx function leads to a rapid and pronounced attenuation of *H2-T3,* followed by a decrease in iCD8α cell number, an increase in macrophage infiltration and activation of pro-inflammatory cascades. These findings suggest a previously unrecognized role for Cdx in intestinal homeostasis through H2-T3-dependent regulation of iCD8α cells.

## Introduction

The intestinal epithelium is home to a variety of cells which function as a barrier and defence against pathogens and conducts the end stages of digestion and absorption of nutrients. Within the small intestine, epithelial structures form finger-like structures, called villi, which project into the intestinal lumen. The colon differs from the small intestine in that it lacks villi and instead exhibits a smooth surface pocketed with crypts. Stem cells near the base of the intestinal crypts divide and displace existing cells along the crypt-villus axis, differentiating into goblet, enterocyte, enteroendocrine, and Paneth cells, the latter of which are unique to the small intestine^1,2^. This process of repopulation must be carefully regulated to maintain intestinal function. Among such regulators are the caudal-related homeodomain transcription factors.

The Cdx homeodomain transcription factors (Cdx1, Cdx2 and Cdx4) are essential for numerous developmental programs^3–8^. Cdx1 and Cdx2 (but not Cdx4) are also expressed in the intestinal epithelium throughout life^5,9^. Cdx1 is distributed in a graded fashion along the proximal–distal intestinal axis, peaking in the distal colon, while Cdx2 expression is maximal in the distal small intestine and proximal colon^5,10^. *Cdx2* null mutant mice are embryonic lethal^8,11^, necessitating derivation of conditional mutant mice for analysis of its post-implantation roles. Using such conditional models, deletion of *Cdx2* using definitive endoderm- or intestinal epithelium-specific Cre transgenes revealed that it is essential for posteriorization of definitive endoderm to an intestinal fate during development and for coordinated differentiation of intestinal cells during embryogenesis and homeostasis in the adult^12–15^. Moreover, while *Cdx1* null mice do not display intestinal defects, loss of *Cdx1* exacerbates the *Cdx2* conditional mutant intestinal phenotype, particularly in the colon^10,14^. This finding is consistent with prior work illustrating functional overlap between Cdx members^7,16^; the term Cdx is therefore used in reference to the sum total of function of all family members in a given context.

While expression analysis has identified a number of intestinal genes regulated by Cdx2, including s*ucrase isomaltase* (*SIS*), *Cadherin 17*, *Dll1,* and *Notch3*^17–19^, the basis by which Cdx regulates intestinal homeostasis is poorly understood. To address this, we used RNA-seq and ChIP-seq, combined with a Cdx conditional mutant model, to identify putative direct Cdx target genes implicated in this process; *H2-T3* was recovered as one such candidate.

*H2-T3* encodes a major histocompatibility complex type Ib protein (MHC-Ib), and is expressed in the thymus and the intestinal epithelium^20,21^. Intestinal intraepithelial lymphocytes (IEL) can be either T-Cell receptor (TCR) positive or negative. TCR negative IEL comprise various subset of lymphocytes, including a group of cells that expresses CD8αα on the surface and CD3 intracellularly (iCD3)^22^. These cells are named innate CD8αα^+^ (iCD8α) and they have the capacity to produce and secrete inflammatory cytokines and chemokines, are capable of engulfing and killing pathogenic bacteria and of processing and presenting antigens to MHC class II-restricted T cells^23–25^. H2-T3 functions to regulate various subtypes of intestinal CD8αα^23^. Activation of iCD8α cells has also been shown to contribute to the development of innate colitis induced by antibodies against CD40^24^.

We have found that that *H2-T3* expression was rapidly lost following *Cdx* deletion in the intestine. *H2-T3* is occupied by Cdx2 in vivo, and is regulated by a Cdx response element in heterologous expression assays, strongly suggesting it is a direct target gene. Attenuation of *H2-T3* expression in Cdx mutants was associated with loss of TCR− CD8αα (iCD8α) lymphocytes specifically in the intestinal epithelium. In addition, iCD8α cells exhibited increased degranulation concomitant with macrophage infiltration and an increase in inflammatory markers in Cdx mutant intestine. Taken together, these findings suggest a novel mechanism by which Cdx impacts intestinal homeostasis.

## Results

### Identification of intestinal Cdx target genes

Cdx1 and Cdx2 play fundamental roles in the intestine presumably through regulation of expression of target genes. To identify such targets, we used RNA-seq, comparing global gene expression between control and *Cdx* mutants, combined with ChIP-seq to identify Cdx-dependent transcription units that were concomitantly occupied by Cdx2. *Cdx1*^−/−^*Cdx2*^F/F^VillinCreERT mice were treated with a single 5 mg dose of tamoxifen by oral gavage between 9 and 12 weeks of age to delete *Cdx2,* yielding animals lacking all Cdx function throughout the intestinal tract^26^; these animals are termed Double Knock Out (DKO) hereafter for simplicity. To control for possible off-target drug effects, control littermates lacking the VillinCreERT transgene were treated with tamoxifen and processed in parallel. Such Cdx1 null mutants do not exhibit any intestinal phenotype and are therefore suitable controls^14^.

**Figure 1:**
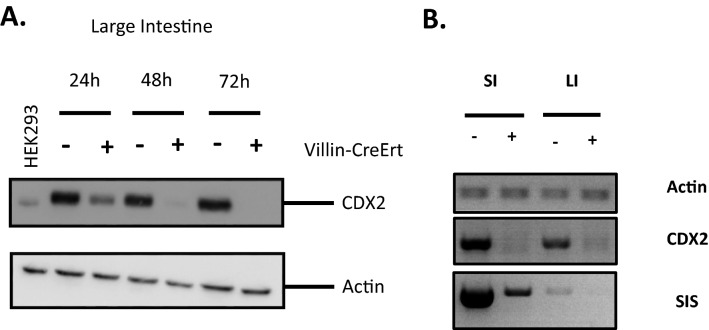
Characterization of *Cdx2* conditional deletion. Mice were treated by oral gavage with 5 mg of tamoxifen dissolved in corn oil and intestinal cells collected at 24, 48 and 72 h. (**A**) Western blot illustrating loss of Cdx2. Actin was used as loading control and HEK293 cell lysates as a positive control for Cdx2. (**B**) RT-PCR analysis, using primers which flank the exon 1 and exon 2 boundary of *Cdx2*, illustrates loss of *Cdx2* and the Cdx-dependent gene *Sucrase Isomaltase* (*SIS*) at 48 h post-tamoxifen treatment.

We first determined the time course of Cre-mediated loss of Cdx2 in DKO mutant intestinal cells by western blot compared to attenuation of expression of *SIS*, a known Cdx intestinal target gene^18^. These analyses revealed significant loss of both Cdx2 protein (Fig. 1A) and *SIS* transcripts (Fig. 1B) 48 h post-deletion. This time point was therefore selected for RNA-seq analysis in order to mitigate secondary effects likely to occur upon protracted loss of Cdx function.

**Figure 2:**
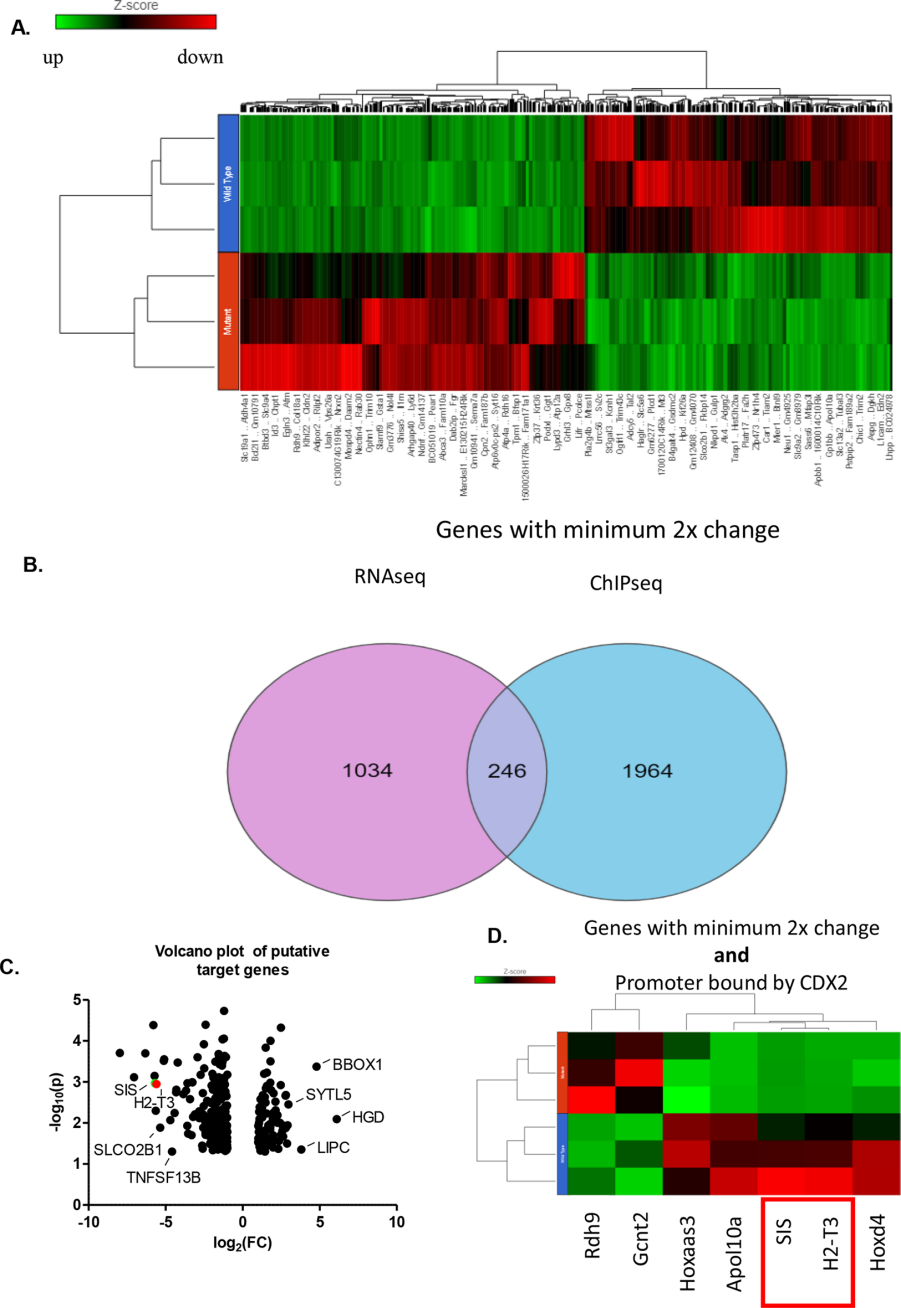
Identification of putative Cdx target genes. (**A**) Heatmap of select Cdx-dependent genes from RNA-seq analysis. A total of 1498 genes were identified with a minimum twofold change in gene expression. Image was genberated using Partek Flow. (**B**) Cdx2-ChIP and RNA-seq identified 246 genes which exhibited both twofold changes in expression and Cdx2 occupancy. Image was generated using Partek Flow. (**C**) Volcano plot of putative target genes highlighting known target genes and putative target genes. Graph was generated using Graphpad Prism 5.0. (**D**) Heat map from RNA-seq analysis of select putative target genes and the known Cdx targets *SIS* and *Hoxd4*. These genes were identified by combining altered expression with Cdx2 binding proximal to the transcriptional start site. Heatmap was generated using Partek Flow.

RNA-seq analysis revealed near-complete loss of *Cdx2* exon 2, as anticipated (data not shown), consistent with western blot analysis. Partec FLOW analysis from biological triplicate samples revealed 1498 genes exhibiting a minimum of twofold change between *Cdx* mutant and control intestinal epithelium with a p-value of ≤ 0.05 (Fig. 2A). Of these genes, 794 exhibited increased expression and 704 showed reduced levels relative to controls (Fig. 2A). Gene ontology (GO) analysis revealed processes associated with immune cell regulation, including complement-related and antigen presentation genes, within the top pathways of transcripts affected by loss of Cdx function (Table 1).

**Table 1:** Gene set enrichment analysis of genes showing twofold expression change following CDX2 deletion and Cdx2 binding in wild type litter mates.

GeneSet	Ratio of proteins in GeneSet	Number of Proteins in GeneSet	*p* value	FDR
Post-translational modification: synthesis of GPI-anchored proteins (R)	0.0081	88	1.11E−16	1.33E−15
Clathrin-mediated endocytosis (R)	0.0108	117	1.11E−16	1.07E−14
Signaling by Rho GTPases (R)	0.0304	330	8.50E−14	4.30E−11
GPCR ligand binding (R)	0.0363	395	1.74E−13	1.06E−11
PPAR signaling pathway (K)	0.0066	72	2.16E−13	8.63E−11
Class I MHC mediated antigen processing and presentation (R)	0.0174	189	2.74E−11	6.19E−09
GPCR downstream signaling (R)	0.0842	915	4.53E−11	1.36E−09
Keratinization (R)	0.0099	108	9.70E−11	2.91E−10

To identify potential direct Cdx targets, we combined the RNA-seq data with the results from ChIP-seq analyses, using Cdx2 occupancy intervals which exhibited a minimum absolute peak summit of 3,000,000 and a *p*-value < 0.05 as determined by Partek-Flow. Peaks were annotated based on their proximity to known genes (Fig. 2B), yielding 246 candidates with occupancy within 2500 bp of transcription start sites (Fig. 2C). *SIS* was recovered in this group, and exhibited a 51 fold reduction in expression, validating the screen (Fig. 2D).

**Figure 3:**
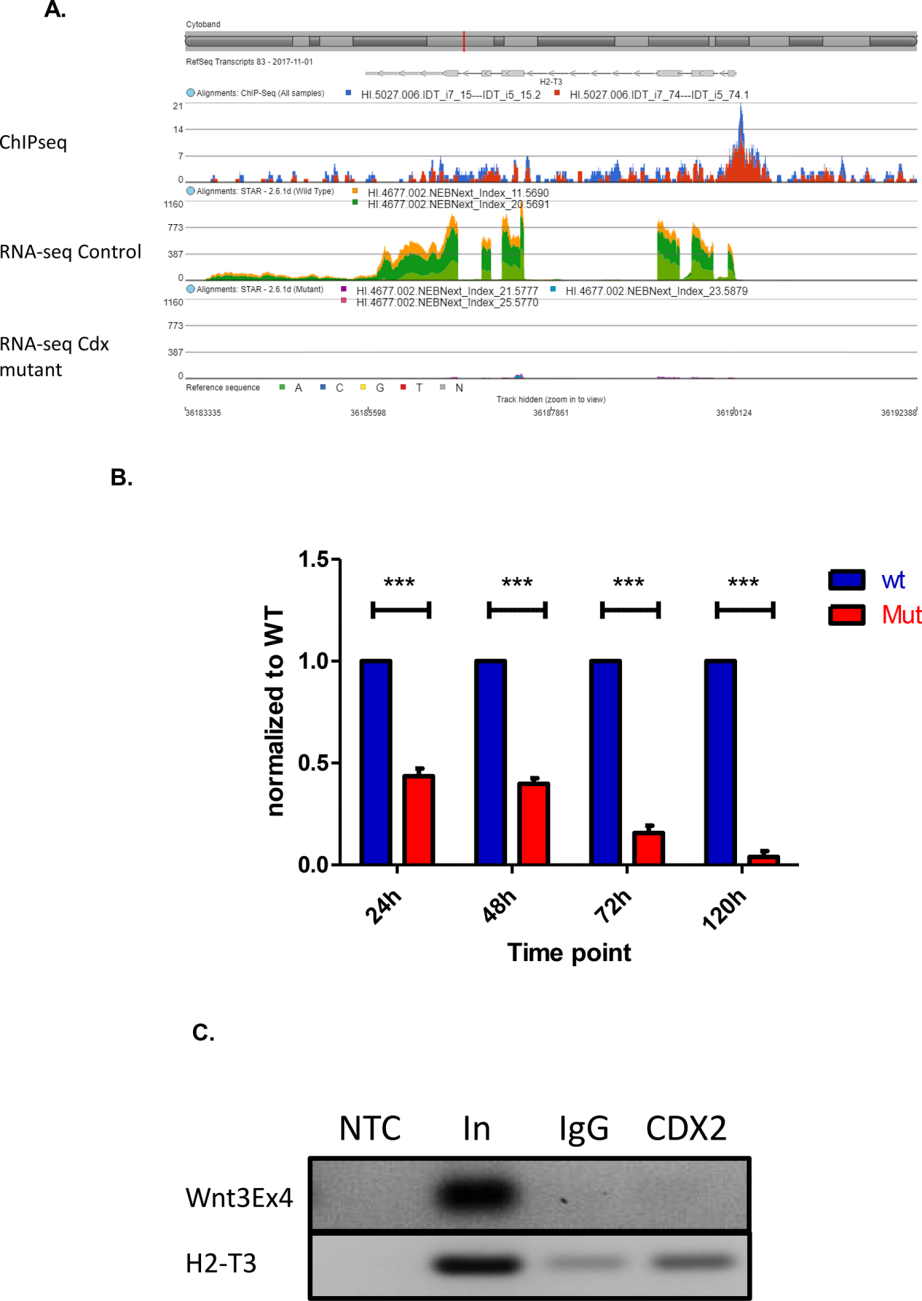
Cdx-dependent regulation of *H2-T3*. (**A**) RNA-seq and ChIP-seq tracks showing reduced expression of *H2-T3* in CDX2 mutant mice with a ChIP-seq peak in the proximal promoter region. Image was generated using Partek Flow. (**B**) RT-qPCR showing loss of *H2-T3* over time following *Cdx2* deletion. Graph and statistical analysis was generated using Graphpad Prism 5.0. Bars represent mean fold change in expression ± SEM from triplicate independent replicates. (***p < 0.001) by ANOVA). (**C**) ChIP-PCR showing binding of Cdx2 to the H2-T3 promoter. IgG immunoprecipitates was used as a negative control.

Among novel candidate targets was *H2-T3,* which encodes an MHC class Ib protein that regulates the development and function of intestinal intraepithelial iCD8α cells (Fig. 2D). RNA-seq revealed that *H2-T3* expression was reduced at 48 h in DKO mutants compared to controls (Fig. 3A), while RT-qPCR analysis from independent biological samples demonstrated loss of *H2-T3* expression as early as 24 h post-tamoxifen treatment, with an approximately 50 fold reduction at 72 h (Fig. 3B). Analysis of the sequences under the Cdx2 ChIP-seq peak at the *H2-T3* locus revealed the sequence TTTATT, a canonical Cdx response element^27^ (CDRE), residing ~ 50 bp upstream of the transcriptional start site of the gene. ChIP-PCR analysis performed using primers flanking this interval confirmed Cdx2 occupancy (Fig. 3C).

**Figure 4:**
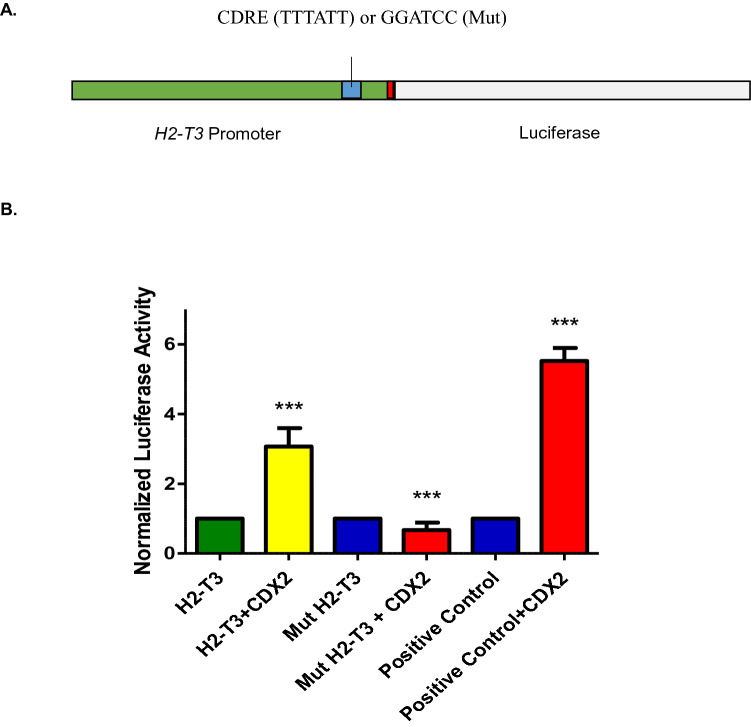
Regulation of *H2-T3* by Cdx2. (**A**) A 1.9 Kb genomic region upstream of the transcriptional start site was used to generate a luciferase reporter (H2-T3) with a wild type (TTTATT) or mutant (GGATCC;Mut) CDRE. Image was drawn using Microsoft PowerPoint software. (**B**) Reporters were transfected into C2BBE1 cells with or without a Cdx2 expression vector and luciferase activity assessed 48 h post-transfection. Graph and statistics were generated using Graphpad Prism 5.0. Bars represent mean luminescence normalized to cells transfected without a Cdx2 expression vector. Significance was calculated by one-way ANOVA (n = 6, ***p < 0.001).

To assess the ability of Cdx to direct expression from the *H2-T3* promoter, and the requirement for the CDRE in this process, a reporter construct containing 1.9 kb of sequences 5′ to the *H2-T3* transcriptional start site was constructed harboring a wild type (TTTATT) or mutant (GGATCC; Mut) Cdx binding motif (Fig. 4A). Co-transfection of the wild type reporter with a *Cdx2* expression vector resulted in a threefold increase in luciferase expression in C2BBE1 cells, and this response was attenuated upon mutation of the CDRE (Fig. 4B). This demonstrates that the *H2-T3* CDRE conveys transcriptional regulation by Cdx2 and, together with the ChIP-seq analysis, is consistent with *H2-T3* being a direct Cdx target gene.

### Cdx impacts iCD8α lymphocyte retention

The primary role of H2-T3 is to serve as a co-factor protein for retention of iCD8α lymphocytes by virtue of its high affinity for the αα homodimer^24^. This interaction also serves to regulate the activation of iCD8α cells, thereby controlling their secretion of various cytokines and granzymes which activate inflammatory responses and act as a chemokine for macrophages^23,28^. We therefore examined the impact of Cdx loss-of function on iCD8α cell retention, their degranulation, and infiltration of macrophage cells. To this end, intestinal cells were isolated from control and DKO mutants and interrogated by flow cytometry for CD45 (as a marker of total immune cells) as well TCRβ, CD8α, CD8β, CD4, F4/80, CD11c and CD206. iCD8α cells were inferred by the lack of expression of TCRβ, positive expression of CD8α and the absence of CD8β. F4/80 was used to identify total macrophage populations, while CD11c and CD206 were used to differentiate between pro- and anti-inflammatory macrophages, respectively, within the F4/80 population.

**Figure 5:**
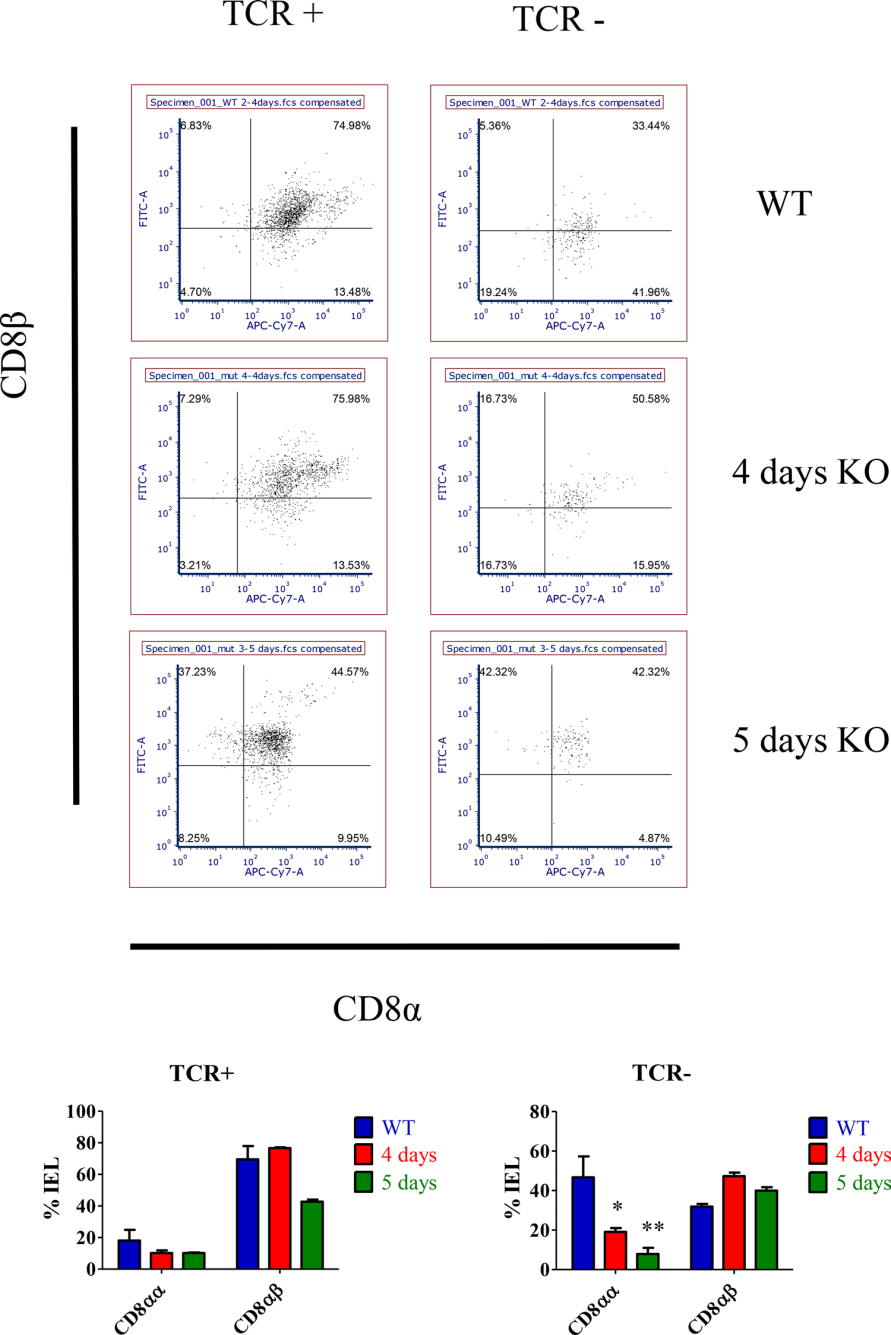
Cdx-dependent immune cell recruitment. Intestinal epithelial and associated cells were collected 4 or 5 days post-tamoxifen treatment and CD45-positive populations analyzed by flow cytometry. Scatter plots of CD45 cells sorted as either TCR+ or TCR- based on TCRβ expression, assessed for CD8α vs CD8β, CD4 vs F4/80, CD11C and CD206 to differentiate inflammatory vs anti-inflammatory macrophages, CD8αα cells were depleted at both time points (lower panels), *p ≤ 0.05; **p < 0.01; ***p < 0.001 by ANOVA.

Cdx DKO mutants exhibited a reduction of iCD8α cells from a relative proportion of 45% total IEL cells to 15% by day four and approximately 1.5% by day five (Fig. 5). A concomitant increase in total macrophages was also seen as early as 4 days after Cdx2 deletion (Supplemental Fig. 1). Within the macrophage population, a threefold increase in the F4/80-CD11c positive cells was observed, consistent with a pro-inflammatory response in mutant intestine. No statistically significant changes were observed in the total CD4+ cell population suggesting that regulatory and helper T-cell populations were unaffected (Supplementary Fig. 1).

**Figure 6:**
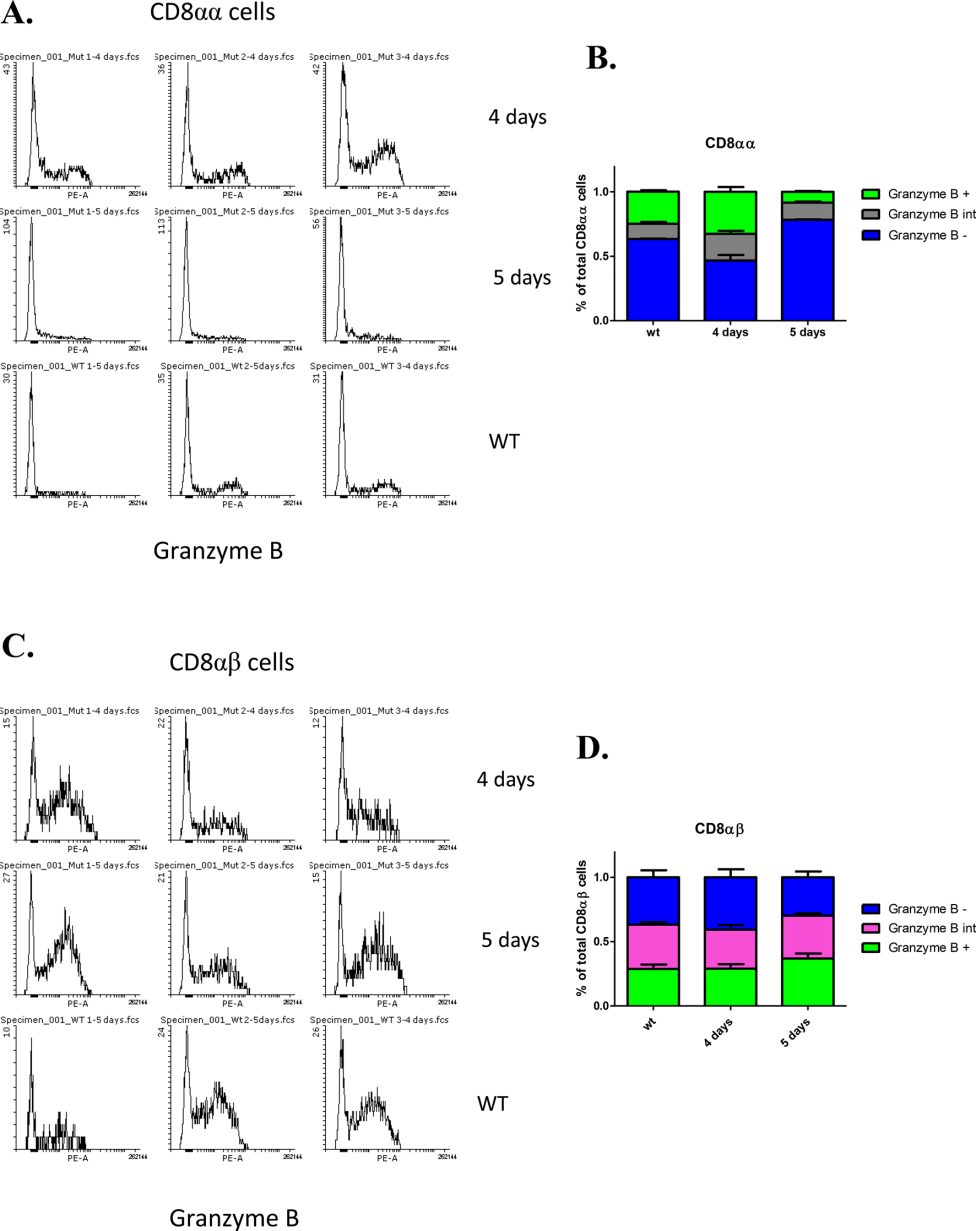
Cdx-dependent CD8αα degranulation. Intestinal cells were isolated 4 or 5 days post-tamoxifen treatment and CD45-positive cells assessed by flow cytometry for CD8α, CD8β and granzyme B. Data is representative of 3 independent biological replicates.

Loss of H2-T3 in IECs has been shown to result in increased iCD8α degranulation leading to a localized inflammatory response including the recruitment of macrophages to the site of reaction^23,24^. Consistent with this, at 4 days post-Cdx2 deletion, we observed that the majority of CD8αα cells were granzyme B-positive. Conversely, we found a near-complete degranulation of CD8αα cells in DKO mutants compared to controls at day 5, while CD8αβ cells retained granzyme B (Fig. 6). While we do note a significant reduction in CD8αβ numbers, this occurs after the loss of iCD8α cells and is likely due to effects unrelated to the attenuation of H2-T3, as the αβ heterodimer is not known to interact with this ligand.

## Discussion

Previous studies have identified Cdx1 and Cdx2 as key regulators of intestinal homeostasis and as context-dependent modifiers of colorectal cancer^10,26,29^, however, the underlying mechanisms by which these outcomes manifest are not fully understood. To address this, we used RNA-seq and ChIP-seq to identify novel Cdx target genes implicated in intestinal homeostasis, leading to our identification of *H2-T3*, which encodes a member of the major histocompatibility complex family, as a Cdx target. We found that Cdx2 occupied the *H2-T3* locus in vivo in an interval which harbors a CDRE necessary to manifest Cdx-dependent transcription in heterologous expression assays. Loss of *H2-T3* in Cdx mutant intestine correlated with reduced numbers of iCD8α cells as well as their degranulation, increased infiltration of macrophages and a pro-inflammatory response. We also observed little change in the TCR+ cell populations which is consistent with prior findings as regards H2-T3 function^21^. Our analysis also showed that macrophage recruitment begins as early 4 days suggesting additional pro-inflammatory mechanisms are evoked by Cdx loss-of-function, the nature of which are presently unknown.

Previous work has shown that Cdx2 deletion in the intestine leads to the recruitment of pro-inflammatory macrophages associated with Cdx-dependent cell non-autonomous signaling which potentiates transformation in a murine model of colorectal cancer (CRC)^30^. Our present observations suggest a possible mechanistic basis for this finding, and are consistent with the effects of disruption of H2-T3, which triggers a loss of the innate CD8αα^+^ lymphocyte population (iCD8α)^21,23^ and subsequent recruitment of pro-inflammatory immune cells^24^.

iCD8α cells play functions similar to innate immune cells. They have the capacity to produce and secrete pro-inflammatory cytokines and chemokines, are capable of engulfing and killing pathogenic bacteria and of processing and presenting antigens to MHC class II-restricted cells^20^. iCD8α are characterized by expression of CD8αα homodimers and negative for expression of TCRα^23^. In addition to presenting H2-T3 to iCD8α cells, IECs also produce and present IL-15 to iCD8α cells^23^. This combination is required for proper development, maintenance and function of iCD8α cells^23^, the mis-regulation of which has been implicated in inflammatory bowel disease (IBD)^24^.

**Figure 7:**
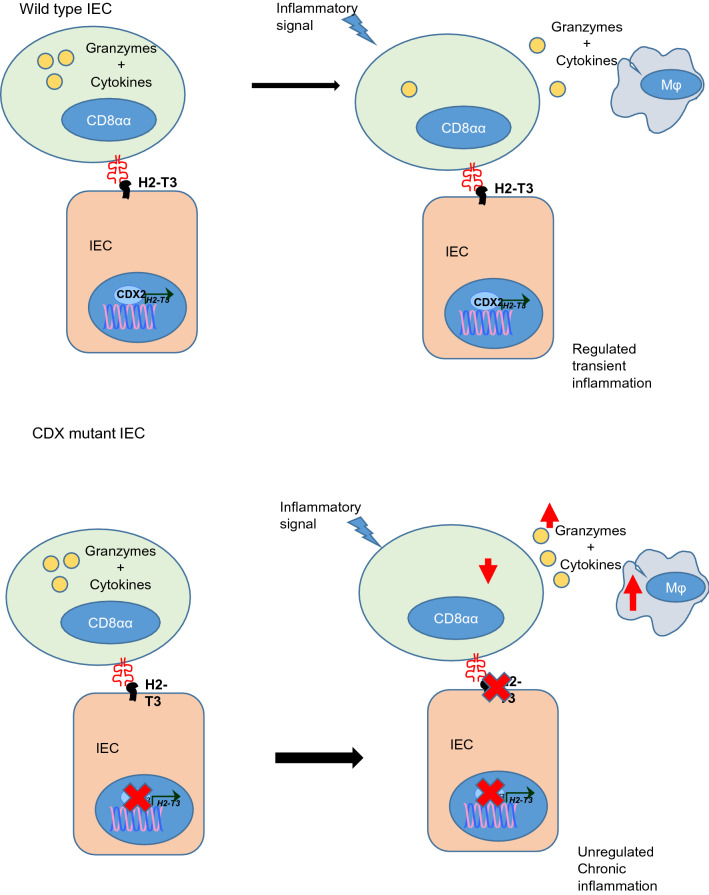
Proposed model of Cdx-dependent regulation of iCD8α lymphocytes. Cdx directly regulates H2-T3 which is required for retention and regulation of iCD8α T cells. The loss of H2-T3 in Cdx DKO mutants results in an increase in secretion of granzyme B from iCD8α cells potentiating the inflammatory response and promoting the migration of macrophages to the epithelial compartment. Image was produced using Microsoft PowerPoint Software.

IBD can occur due to aberrant immune cell activity and may involve a T-cell autoimmune disorder^31,32^. This can manifest as several forms, including over-activity of innate T-cells or macrophage populations, either of which can result in a chronic inflammatory response^33–35^. In this regard, loss of expression of *CDX2* has been associated with the development of ulcerative colitis^36–38^. While the basis underlying this observation is unresolved, increased TNFα secretion, triggered by activation of pro-inflammatory pathways, has been association with reduced expression of CDX2 in IBD patients^36,37^. Our present results suggest an additional pathway whereby attenuation of CDX2 leads to subsequent loss of *H2-T3* and further promotes an inflammatory state through dysregulation and degranulation of iCD8α cells (Fig. 7). Also consistent with this model is the finding that activation of iCD8α cells contributes to the development of innate colitis induced by antibodies against CD40^24^, further supporting a link between Cdx, iCD8α cells and IBD. Although human iCD8α cells have been identified in the intestinal tract and may play a similar role as in the mouse, a human homologue of H2-T3 has not yet been reported.

In summary, we have revealed a novel mechanism by which Cdx can modulate homeostasis in intestinal epithelial cells through regulating local immune responses. Chronic inflammatory responses have been implicated in a variety of disorders including IBD and CRC, both of which are impacted by Cdx. While *Cdx2* germ line inactivation is unlikely to underlie such diseases, due to lethality, somatic mutations, altered epigenetic regulation of *Cdx2* expression or mutation of Cdx-dependent regulatory elements in critical target gene *loci* may contribute to certain pathologies.

## Materials/methods

### Mouse strains

Mice were bred to generate *Cdx1*^−/−^*Cdx2*^F/F^VillinCreERT animals as previously described1^26^. Animals were treated with a single 5 mg dose of tamoxifen by oral gavage between 9 and 12 weeks of age to delete *Cdx2,* yielding animals lacking Cdx1 and Cdx2 throughout the intestinal tract and termed Double Knock Out (DKO) for simplicity. Control littermates lacking VillinCreERT were treated in parallel to control for off-target effects of tamoxifen. Animals were maintained in accordance with established guidelines from the Canadian Council on Animal Care as prescribed by the animal care and veterinary services at the University of Ottawa. Experimental protocols were approved by the animal care and veterinary services at the University of Ottawa. This study was carried out in compliance with ARRIVE guidelines.

### Intestinal epithelial cell isolation

Intestinal epithelial cell isolates were enriched for crypt cells and as described previously^39^. Briefly, from 1 to 5 days post-tamoxifen treatment, mice were euthanized, colons dissected in cold Phosphate Buffered Saline (PBS), opened longitudinally and rinsed with PBS. Intestines were then incubated in 5 ml of cold PBS 1.5 mM in EDTA for 15 min and tissue vortexed to dislodge epithelial cells. This process was repeated for a total of 6 cycles, with the 5th and 6th isolates, which were enriched in crypt cells, retained for analysis.

### RNA-sequencing (RNA-seq)

For RNA-seq, intestinal cells were isolated as above 48 h post-treatment with 5 mg of tamoxifen from 3 mutant mice and 3 littermate controls. RNA was extracted using RNeasy as per the manufactures directions (Qiagen). RNA quality was assessed using an Agilent Bioanalyzer, poly-A + mRNA was enriched and fragmented, and libraries generated with cDNA which underwent rend tail repair and subsequent dA tailing followed by adaptor ligation. Samples with a minimum RNA integrity of 8, assessed by Bioanalyzer, were used to generate cDNA libraries which were sequenced on an Illumina HiSeq4000 system to a minimum of approximately 50 million reads per sample.

### Chromatin immunoprecipitation-sequencing (ChIP-seq)

ChIP-seq analysis was conducted as previously described^40^ from two independent biological replicates. Briefly, nuclei were isolated by lysing intestinal cells in 4% Tween PBS and chromatin sheared by sonication to generate DNA fragments averaging 500 bp, verified by gel electrophoresis. Cdx2-associated chromatin was isolated using Dynabeads conjugated to an anti-*Cdx2* antibody. Protein-DNA complexes were isolated, reverse crosslinked by heating at 65 °C for 18 h and DNA purified using Qiagen PCR purification kits as per the manufactures direction. Sequencing was conducted on an Illumina Nextgen sequencing column to 24–34 million reads per sample.

RNA-seq and ChIP-seq in silico analyses were performed using Partek FLOW software. To assign potential direct *Cdx2* targets, changes in transcript abundance between wild type and *Cdx* mutant intestinal cells were correlated with Cdx2 genome occupancy. Binding motif analysis was performed to identify putative Cdx2 binding elements (CDRE) under ChIP peaks, and targets further validated by ChIP-PCR using primers flanking such CDREs (Table 1) as previously described^40^. Negative controls included PCR from IgG precipitates or primers flanking regions of chromosomal DNA negative for Cdx2 binding.

### Promoter analysis

Cell-based reporter assays were used to assess Cdx2-dependent regulation of the putative CDRE identified within the *H2-T3* promoter. The reporter was generated from a 1.9 kb PCR amplicon of genomic DNA immediately 5′ to the *H2-T3* transcriptional start site. This interval was then cloned into the pXP-2 luciferase plasmid and verified by sequencing. Site-directed mutagenesis was used to generate an identical reporter construct, mutating the *H2-T3* CDRE (TTTATT) to a Bam HI recognition sequence (GGATCC).

C2BBE-1 and HEK293T cells (ATCC) were cultured in DMEM (Hyclone) supplemented with 5% FBS. Transferrin (0.01 mg/ml) was also included for C2BBE-1 cells. Cells were maintained at 37 °C in either 5% CO_2_ (for HEK293T cells) or 10% CO_2_ (for C2BBE-1 cells). Cultures were transfected with reporter constructs (750 ng) with a *Cdx2* expression vector or empty expression vector control (250 ng) using Attractine transfection reagent (Qiagen) as per the manufacturer’s directions. Cells were collected 48 h post-transfection for analysis of promoter activity using a Promega luciferase assay kit as per the manufacturer’s direction. Luciferase activity was standardized to total protein content.

### Flow cytometry

IEL cells were purified using a Percoll gradient as previously described^41^. An aliquot was subjected to heating at 65 °C for 20 min to induce cell death. Dead cells were pooled with live cells to serve as an internal control for live/dead staining, which was performed using zombie yellow (Biolegend) as per the manufacturers’ directions. Cells were then washed twice in 2 ml of cold PBS for 5 min, fixed in 4% paraformaldehyde for 20 min at room temperature and pre-treated with anti-Fc antibody for 1 h and subsequently for 2 h with fluorophore-conjugated antibodies. Cells were then washed to remove excess antibody and analyzed by flow cytometry, gating to count live CD45+ cells and analyzed for the abundance of TCR+ , TCR− and sub population of CD8+ cells, F4/80 macrophages or M1/M2 macrophages (CD11c or CD206; see Supplementary Table 2).

### Quantitative PCR (RT-qPCR)

RNA was isolated from intestinal epithelial cells, cDNA generated by standard means and amplified by PCR using primers described in Supplementary Table 1. Thermo-cycler conditions were as follows: 95 °C for 5 min (Hot start), 95 °C for 30 s, 30 s for annealing (see Supplementary Table 1for annealing conditions), 72 °C for 30 s/kb for a total of 29 cycles, determined experimentally to fall with the linear phase of amplification. Products were resolved by agarose gel electrophoresis and quantified by densitometry using Alpha-ease software, standardized to β-actin expression.

For qPCR, 5 μl of cDNA was amplified for 40 cycles with SYBR Green (Go-Taq) in triplicate using a Bio-Rad CFX qPCR machine, followed by dissociation curve analysis. Cycling was 95 °C for 3 min, annealing at 58 °C for 30 s and elongation of the primers at 60 °C for 30 s. Data was analysed using the Bi-Rad CFX manager software and gene expression was expressed relative to *GAPDH* by (ΔCt) then standardized to control expression (ΔΔCt).

### Western blot analysis

Cells from either intestinal epithelium or tissue culture were collected and lysed in RIPA buffer. Protein was quantified by Bradford assay as per the manufacturer’s directions (Bio-Rad), lysates resolved by polyacrylamide gel electrophoresis and transferred to PVDF membranes. Membranes were blocked using 5% skim milk in Tris-buffered Saline + 0.1% Tween (TBST) and incubated with primary antibodies against Cdx2^16^ or β-Actin (Santa Cruz) (diluted to 1:1,000 in 5% skim milk in TBST). Membranes were washed in TBST three times for 5 min each and incubated with HRP-conjugated secondary antibody (Santa Cruz Biotechnology, 1:10,000 in 5% skim milk in TBST). Following washing with TBST, reactivity was revealed by luminescence using GE Forteza ECL solution, imaged with a GE-Gel Doc XR + System and analyzed using Alpha-ease image analysis software.

### Statistical analysis

Densitometry analysis was conducted using GraphPad Prizm. Flow cytometry data was initially analyzed using Flowing 2.0 software, and subsequently using GraphPad Prism for T-tests, 1-way or 2-way ANOVA with Tukey post-hoc tests as appropriate.

## Supplementary Information

**Supplementary Information**.
