# Supplementary material for: Cdx2 regulates immune cell infiltration in the intestine
Source: Sci Rep. 2021 Aug 4;11:15841. doi: 10.1038/s41598-021-95412-w (PMC8339112; doi:10.1038/s41598-021-95412-w)

Supplementary Table 1 List of PCR primers used.

Gene Name	Primer sequence	Ta
Actin RT-PCR	F: CCCTAAGGCCAACCGTGAAA<br>R: GTCCTATGGGAGAACGGCAG	54 ˚C
Cdx2 RT-PCR	F: AGCCAAGTGAAAACCAGGACAA<br>R: TGCAAGGAGGTCACAGGACT	54 ˚C
SIS RT-CRP	F: GCGTAGAGAGAGGCTGCATA<br>R: GCCCGTTGCCAATTTTTGAG	53 ˚C
H2-T3 Q-PCR	F: CAATGGTGGAAACGGAGACG<br>R: GAGTGCCTCAGATTCAGCCC	58 ˚C
GAPDH Q-PCR	F: GGCCGGTGCTGAGTATGTCG<br>R: TTCAAGTGGGCCCCGGCCTT	58 ˚C
H2-T3 Promoter region	F: GGCTGTTTCCCTCCTCATCC<br>R:AAAGGCCTTGCACTCCTGTT	52 ˚C

Supplementary Table 2 list of flow cytometry antibodies

Target Protein	Catalogue number<br><br>(all from Bio-Legend)
Fc blocking	101319
CD45	103125
CD8α	100713
CD4	100515
CD335	137607
CD11c	117309
F4/80	123109
Granzyme B	372207
CD8β	126605
Cd206	141703
Cd11b	101205

Supplementary Figure 1. Cdx-dependent immune cell recruitment. Intestinal epithelial and associated cells were collected 4 or 5 days post-tamoxifen treatment and CD45-positive populations analyzed by flow cytometry. **A.** Scatter plots of CD45 cells, assessed for CD8α vs CD8β, CD4 vs F4/80, CD11C and CD206 to differentiate inflammatory vs anti-inflammatory macrophages, **B.** Specific cell populations were compared as a percentage of total immune cells. CD8αα cells were depleted at both time points, while inflammatory macrophage populations were increased 4 days post treatment. * $p \leq 0.05$, ** $p<0.01$, ***$p<0.001$ by ANOVA.

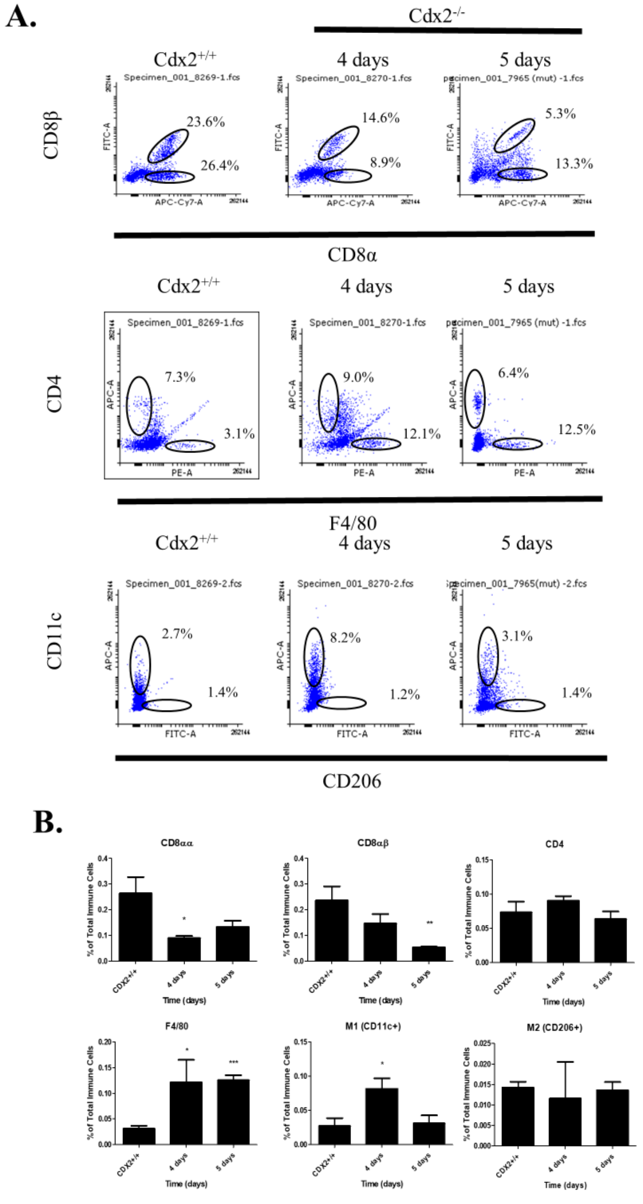

Supplementary Figure 2. Full gel images for CDX2 expression in knock out mice by western blot and RT-PCR

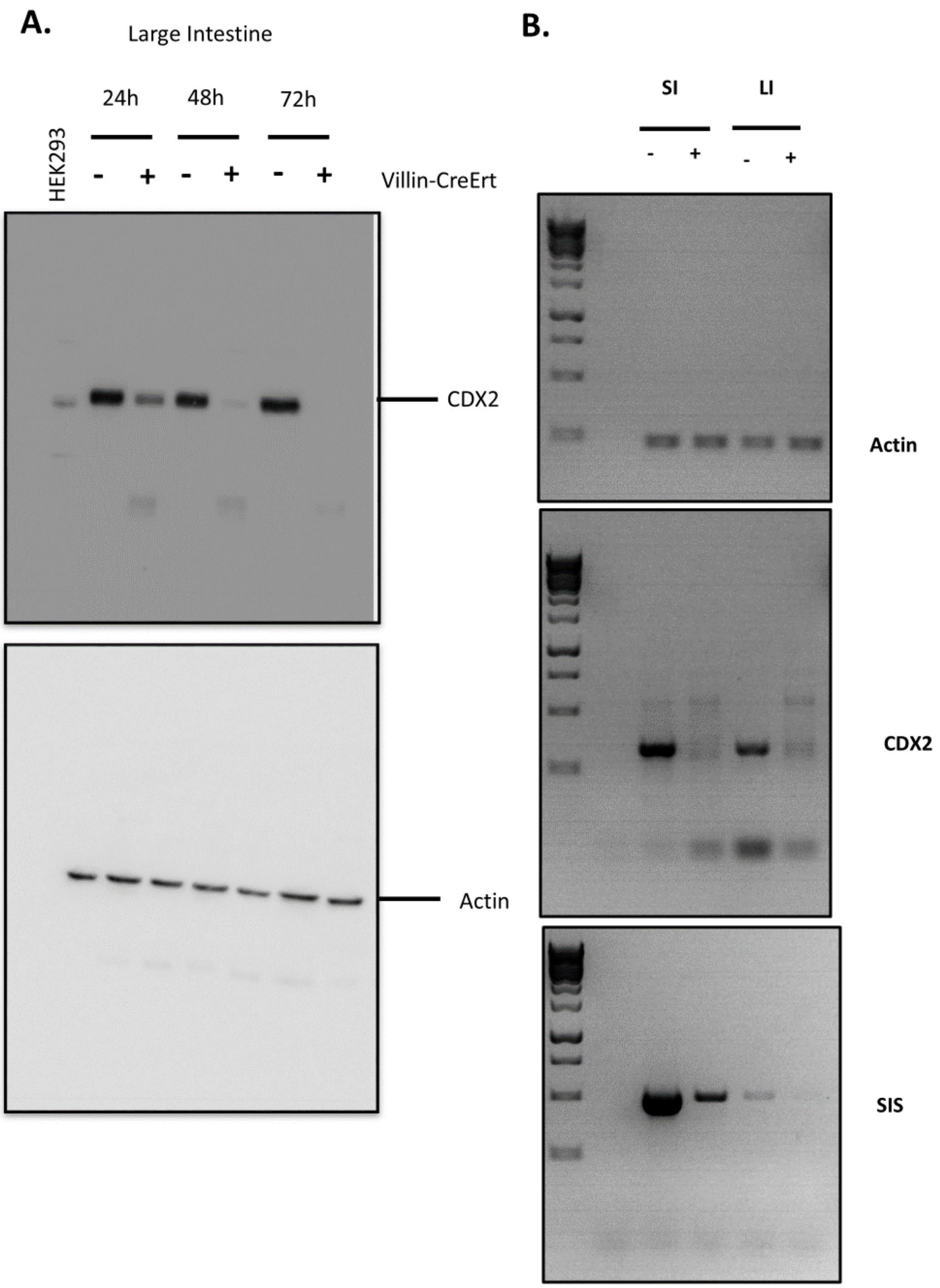

Supplementary Figure 3. Full gel images for ChIP data of CDX2 pulldown.

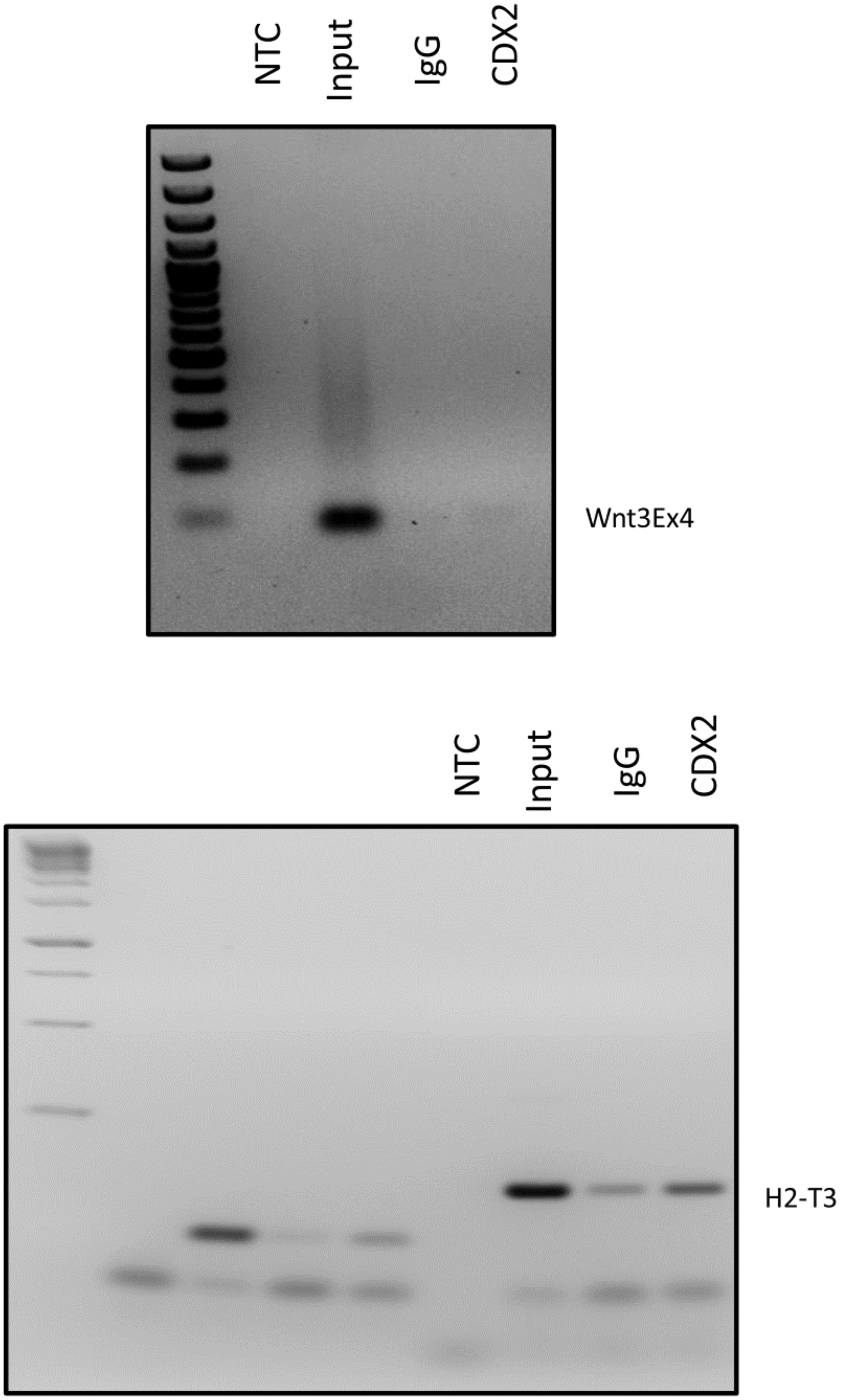

Supplement: Supplementary file 1 — Supplementary Information. [file 41598_2021_95412_MOESM1_ESM.pdf]
